# Indocyanine Green as a Marker for Nose-to-Brain Delivery Pathways, Brain Distribution, and PLGA Nanoparticle Efficiency

**DOI:** 10.3390/ijms27041782

**Published:** 2026-02-12

**Authors:** Milena Mishonova, Lea Koceva, Bissera Pilicheva, Plamen Zagorchev, Neli Raikova, Mitko Mladenov, Rossitza Konakchieva, Hristo Gagov, Iliyana Sazdova

**Affiliations:** 1 Department of Animal and Human Physiology, Faculty of Biology, Sofia University “St. Kliment Ohridski”, 8 Dragan Tsankov Blvd., 1164 Sofia, Bulgaria; mmishonova@biofac.uni-sofia.bg (M.M.); lealk@uni-sofia.bg (L.K.); neliraikova@biofac.uni-sofia.bg (N.R.); i.sazdova@biofac.uni-sofia.bg (I.S.); 2 Institute of Neurobiology, Bulgarian Academy of Sciences, Acad. G. Bonchev Str., Bl. 23, 1113 Sofia, Bulgaria; 3 Faculty of Pharmacy, Medical University of Pleven, 1 St. Kliment Ohridski Str., 5800 Pleven, Bulgaria; bisera.pilicheva@mu-pleven.bg (B.P.); plamen.zagorchev@mu-pleven.bg (P.Z.); 4 Institute of Biology, Faculty of Natural Sciences and Mathematics, Ss. Cyril and Methodius University, 1000 Skopje, North Macedonia; mitkom@pmf.ukim.mk; 5 Department of Fundamental and Applied Physiology, Russian State Medical University, 117997 Moscow, Russia; 6 Department of Cell and Developmental Biology, Faculty of Biology, Sofia University “St. Kliment Ohridski”, 1164 Sofia, Bulgaria; r.konakchieva@biofac.uni-sofia.bg

**Keywords:** near-infrared, olfactory bulb, nasal mucosa, trigeminal nerve, brainstem, PLGA nanoparticles, nanocomposite microparticles, indocyanine green, nose-to-brain

## Abstract

This study aims to assess the rate and duration of rat brain retention after a single intranasal administration of indocyanine green (ICG) as an aqueous solution or encapsulated in poly(D,L-lactide-co-glycolide) (PLGA) nanoparticles. Near-infrared fluorescence emission of ICG from the brain and visceral organs was measured at 1, 4, and 24 h, as well as at 1 and 2 weeks after administration. It was observed that both ICG formulations stained the olfactory bulbs and brainstem, the latter mainly in the basolateral region of the pons. Reduced staining was observed on day 7 after treatment, and the signal remains detectable on day 14. Additionally, while emission from ICG-labeled brains in water decreased after two weeks compared to day 7, in ICG-loaded nanoparticles, the emission was significantly higher on day 14. It is concluded that ICG is transported into the brain via both nose-to-brain delivery pathways—through and along olfactory or trigeminal nerves—and that ICG is a useful dye for in vivo studies due to its long-lasting emission and low toxicity. Furthermore, the suggested penetration of ICG-encapsulated PLGA nanoparticles via these transport mechanisms makes them a useful carrier for brain delivery of substances that are rapidly eliminated from circulation or do not cross the blood–brain barrier.

## 1. Introduction

Since the pioneering work of William Frey (1991) [1], numerous studies have investigated the potential of nose-to-brain delivery mechanisms for administering neurological agents to study, protect, support, and restore neuronal functions, thereby bypassing systemic circulation and the blood–brain barrier [2,3,4]. Intranasal administration has been used not only to deliver small molecules with molecular weights up to approximately 1000 Daltons, which readily penetrate the nasal mucosa and are rapidly transported into the brain, but also to deliver larger biomolecules such as insulin, oligonucleotides, monoclonal antibodies, and basic fibroblast growth factor [2,4]. The recent surge in nanostructure-based applications has highlighted nanoparticles, liposomes, and related nano- and microcarriers as promising platforms for the targeted delivery of neuroprotective and therapeutic agents to the central nervous system (CNS) [5,6,7]. Additionally, the intranasal method for non-invasive delivery of neuronal stem cells to the CNS seems promising for the treatment of cerebral palsy and Parkinson’s disease [8,9,10]. Therefore, the advantages of nose-to-brain administration, such as its non-invasive nature and higher efficiency for CNS delivery compared with oral or intravenous application, are expected to draw significant attention to its future use in the treatment of both chronic and acute neuropathological conditions [4,11].

To enhance drug absorption, delivery systems engineered at micro- and nanoscale dimensions are increasingly developed. Particle retention within the nasal cavity is influenced both by the anatomical structure of the cavity and particle-specific properties, including size, shape, and density [12]. Experimental evidence indicates that inhaled particles larger than 20 μm predominantly deposit in the nasal vestibule. In contrast, particles smaller than 5 μm, as well as nanoscale structures, tend to follow the airstream and are expelled during exhalation [13,14]. During normal inhalation, particles with diameters of approximately 10 μm can reach and deposit within the olfactory region [15]. Substantial evidence indicates that microparticles enhance drug delivery to the CNS primarily by prolonging residence time on the nasal mucosa; however, their relatively large size limits their ability to penetrate the nasal epithelium [16]. Instead, they continuously release the active substance onto the mucosal surface, from which it is absorbed by passive diffusion. Beyond particle size, residence time within the nasal cavity is a key determinant of absorption efficiency. The incorporation of mucoadhesive polymers into carrier systems further prolongs contact with the nasal epithelium, thereby facilitating improved drug uptake. Unlike microparticles, nanoparticles can penetrate phospholipid membranes and deliver drug molecules to specific target sites, protecting them from enzymatic or chemical degradation. However, their small size makes it difficult to retain them within the olfactory region of the nasal cavity, increasing the likelihood of their inhalation into the lungs [17]. A promising approach to overcoming these limitations in nose-to-brain delivery is the development of composite systems integrating nanoparticles and microparticles into a single structure, such as nanocomposite microparticles. These composite carriers not only combine the advantages of both components but also exhibit emergent properties that neither the nanoparticles nor the microparticles possess individually.

Drug carriers can be selected from a wide range of natural, semi-synthetic, and synthetic polymers. Key selection criteria include biocompatibility, biodegradability, low toxicity, mucoadhesiveness, good coating capacity, and cost-effectiveness. Polylactic-co-glycolic acid (PLGA), an aliphatic polyester formed by the copolymerization of lactic and glycolic acids, is widely regarded as an optimal polymer for nanoparticle-based nose-to-brain delivery owing to its excellent biocompatibility, safety profile, and regulatory approval [18]. Its degradation products, lactic and glycolic acids, are endogenous metabolites, ensuring nontoxic elimination and good tolerability by the sensitive nasal mucosa [19]. A key advantage of PLGA over other polymeric carriers is its highly tunable degradation profile, which can be precisely adjusted through the lactide-to-glycolide ratio and molecular weight, enabling predictable and sustained drug release [20]. PLGA nanoparticles exhibit high structural stability and versatile drug-loading capacity, accommodating both hydrophilic and hydrophobic molecules while shielding them from enzymatic degradation within the nasal cavity [21,22]. The polymer is also compatible with scalable manufacturing techniques and permits surface modification to enhance mucoadhesion, mucus penetration, or neuronal uptake [23]. Collectively, these properties establish PLGA as a robust and well-characterized platform for nanoparticles intended for efficient drug transport along the olfactory and trigeminal pathways. Numerous studies have further shown improved performance of PLGA-based systems when combined with hydrophilic polymers [24,25,26].

Indocyanine green (ICG) is a widely used water-soluble fluorescent dye with low toxicity in humans [27]. The near-infrared (NIR) emission of ICG, within the wavelength range of 800–830 nm, makes it a suitable contrast agent for in vivo, ex vivo, and in vitro measurements, due to its deep tissue penetration and efficient emission in biological structures. Importantly, this emission range does not interfere with the fluorescence of cells, including erythrocytes, whose emission is in the 500–600 nm range [27,28,29]. A previous study examined the nose-to-brain delivery of ICG using nanostructured lipid carriers, and brain penetration was monitored in vivo via fluorescence emission at 840 nm at several time points up to 24 h post-administration [30]. Although this work provided compelling evidence for effective cerebral delivery of ICG, it did not investigate the specific intracerebral localization of the dye or elucidate the transport pathways involved.

The present study aimed to compare the nasal penetration and the efficiency of nose-to-brain delivery of ICG in rats when administered either as an aqueous solution or encapsulated in PLGA/fucoidan nanocomposite microparticles, and to identify the specific brain regions where it accumulates.

## 2. Results

### 2.1. Rat Nostrils NIR Fluorescence

**Figure 1:**
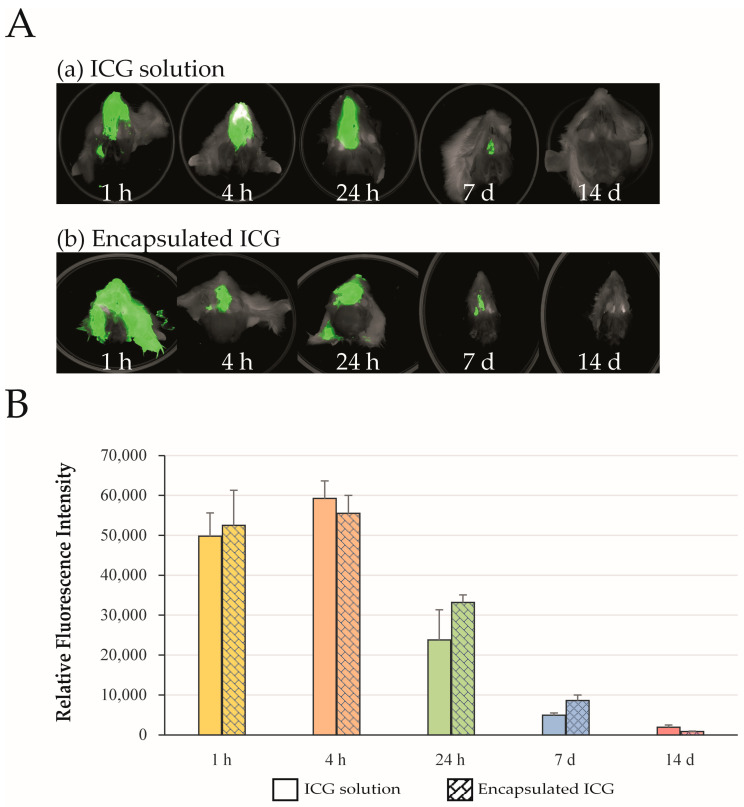
Nasal cavity. (**A**) Ex vivo NIR bioimaging of the nasal cavity after application of ICG solution (panel (**a**), *n* = 6) or ICG-encapsulated PLGA nanoparticles (panel (**b**), *n* = 6) at 1 h, 4 h, 24 h, 7, and 14 days. (**B**) RFI values of ICG fluorescence in the rat nasal cavity obtained at the indicated time points after the administration of ICG as an aqueous solution (colored columns, *n* = 6) or as ICG-loaded nanocomposite microparticles (patterned columns, *n* = 6). Different colors represent the time points at which the brain was extracted following application. Data are presented as mean ± SEM. No statistically significant differences between groups were observed (Student’s *t*-test, all *p* > 0.05).

Ex vivo near-infrared imaging revealed detectable ICG fluorescence in the rat nasal cavity at 1 h, 4 h, 24 h, 7 days, and 14 days following a single intranasal administration (Figure 1A). Figure 1A shows representative NIR emissions of the nostrils of ICG applied as an aqueous solution (**a**) or as ICG-encapsulated PLGA nanoparticles (**b**). The strength of the signal was measured as the relative fluorescence intensity (RFI) of the ICG emission at 800 nm wavelength. Both the aqueous and nanoparticle-encapsulated ICG produced persistent fluorescent signals throughout the observation period. Quantitative analysis of RFI showed no statistically significant differences between the aqueous and microparticle formulations at any time point during the study period (Figure 1B; *n* = 6 per group; all *p* > 0.05). Given the sample size and observed variability, the study was powered to detect only large differences in nasal retention; therefore, smaller biologically relevant differences cannot be excluded. Within these limits, the results indicate broadly comparable nasal retention profiles for both formulations, suggesting that encapsulation of ICG in PLGA nanoparticles does not markedly alter its initial residence on the nasal mucosa compared with the aqueous formulation.

### 2.2. NIR Fluorescence from the Rat Brain

#### 2.2.1. Whole Rat Brain NIR Emission

**Figure 2:**
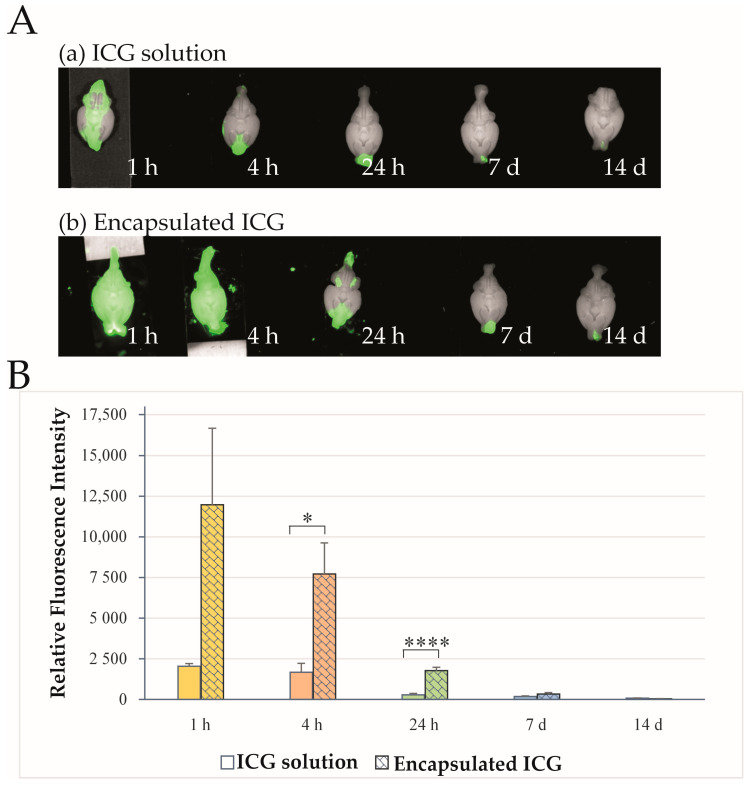
Ex vivo NIR fluorescence images of rat brains at 1 h, 4 h, 24 h, 7 days, and 14 days after intranasal administration of ICG. (**A**) Representative NIR fluorescence images after application of ICG as an aqueous solution (panel (**a**), *n* = 6) or as ICG-encapsulated PLGA nanoparticles (panel (**b**), *n* = 6) at the same time points. (**B**) RFI values of ICG fluorescence in the rat brain obtained at the corresponding time points after the administration of ICG as an aqueous solution (colored columns, *n* = 6), and of ICG-encapsulated PLGA nanoparticles (patterned columns, *n* = 6). Different colors indicate the time of brain extraction after administration. Data are presented as mean ± SEM (*n* = 6). * *p* < 0.05, **** *p* < 0.0001.

Representative NIR fluorescence images of rat brains in ventral view obtained after intranasal administration of ICG are shown in Figure 2A. Fluorescent emission was measured at 1 h, 4 h, 24 h, and 7 or 14 days after a single administration of ICG as an aqueous solution (a) or as ICG-loaded nanocomposite microparticles (b). A stronger signal was typically observed in the right olfactory bulb, corresponding to the side of intranasal administration (right nostril). Quantitative analysis of RFI demonstrated significantly higher whole-brain fluorescence intensity at 4 and 24 h in animals treated with ICG-encapsulated PLGA nanoparticles compared with those treated with aqueous ICG (Figure 2B; *p* = 0.0196, *n* = 6 at 4 h; *p* = 0.000044, *n* = 6 at 24 h). At 1 h and 7 days after administration, whole-brain RFI values were higher in the nanoparticle-treated group; however, the differences were not statistically significant (1 h: *p* = 0.139, *n* = 5; 7 days: *p* = 0.145, *n* = 6). At day 14, the aqueous ICG-treated group showed higher signal intensity, but this difference was also not statistically significant (*p* = 0.0556, *n* = 5).

#### 2.2.2. NIR Emission from a Particular Rat Brain Area

**Figure 3:**
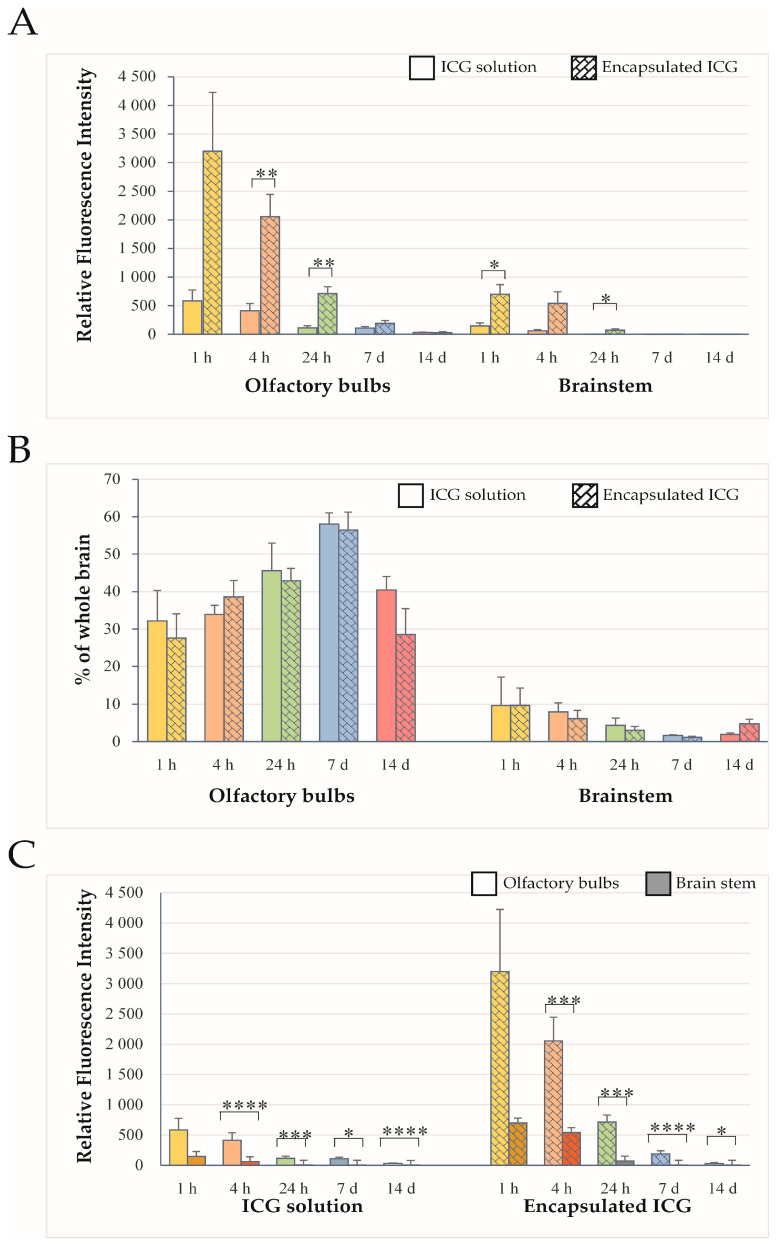
Regional brain distribution of intranasally administered ICG. (**A**) Relative fluorescence intensity (RFI) of ICG measured in the olfactory bulb (OB) and brainstem at 1 h, 4 h, 24 h, 7 d, and 14 d after intranasal administration of ICG as an aqueous solution (solid-colored bars) or PLGA-encapsulated formulation (patterned bars). Statistical comparisons in panel A are between the two formulations at each time point. (**B**) OB and brainstem RFI expressed as a percentage of the total whole-brain fluorescent signal. Statistical comparisons in panel B are between formulations at the corresponding time points. (**C**) Direct comparison of OB and brainstem RFI values over time following administration of aqueous ICG (**left**) and ICG-encapsulated PLGA nanoparticles (**right**), with data presented by brain region within each treatment group. Statistical comparisons in panel C are between time points within the same treatment group. Data are presented as mean ± SEM (*n* = 6). * *p* < 0.05; ** *p* < 0.01; *** *p* < 0.001; **** *p* < 0.0001. ICG-encapsulated PLGA nanoparticles.

NIR fluorescence originating from the olfactory bulb (OB) and brainstem was quantified at 1 h, 4 h, 24 h, 7 days, and 14 days after intranasal administration of ICG either as an aqueous solution or encapsulated in ICG-encapsulated PLGA nanoparticles (Figure 3). For both formulations, RFI values in the OB were consistently higher than those measured in the brainstem at early time points, particularly within the first 24 h after administration (Figure 3A). Quantitative comparison of OB and brainstem fluorescence expressed as a percentage of the total whole-brain signal revealed no significant differences between the aqueous and nanoparticle formulations (Figure 3B; *p* > 0.05), indicating that both transport pathways contributed proportionally to total brain uptake. Comparison of RFI values demonstrated significantly higher fluorescence intensity in the OB compared with the brainstem at early time points following intranasal administration (Figure 3C; *p* < 0.05–0.0001), irrespective of the formulation used. This pattern persisted throughout the observation period, suggesting preferential propagation of ICG along the olfactory pathway relative to the trigeminal pathway.

**Figure 4:**
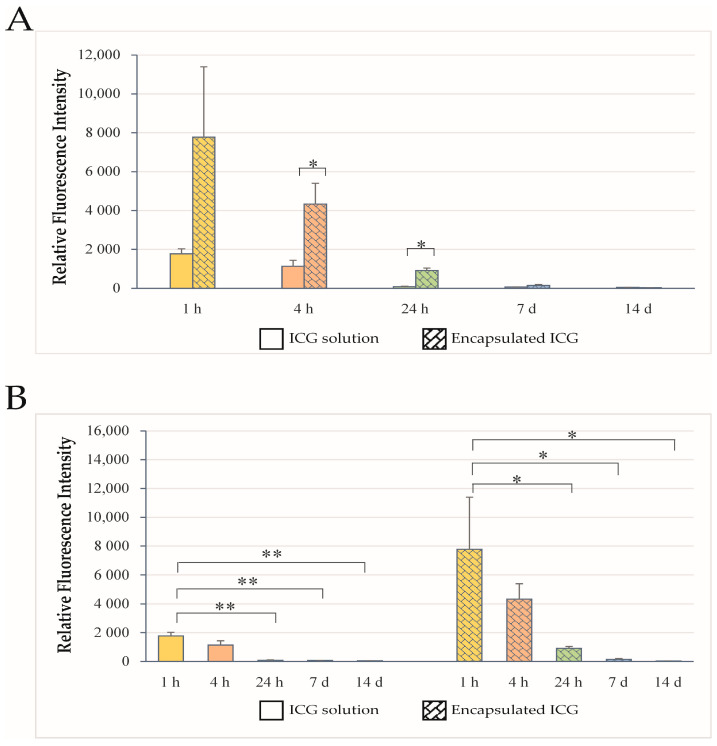
Time-dependent RFI values corresponding to residual fluorescence of the whole brain, excluding olfactory bulb and brainstem signals, following intranasal administration of ICG (**A**) as an aqueous solution (solid columns) or encapsulated in ICG-encapsulated PLGA nanoparticles (pattern-colored columns), presented as time after application. Statistical analyses are between the treatment groups at the respective time intervals. (**B**) Time-dependent changes in the residual signal for each application as an aqueous solution (**left**) or as ICG-encapsulated PLGA nanoparticles (**right**) presented by application. Statistical analyses are between time intervals within each treatment group. Data are presented as mean ± SEM (*n* = 6). * *p* < 0.05; ** *p* < 0.01.

To evaluate the distribution of ICG within brain regions other than the olfactory bulbs and brainstem, the RFI values of the OB and brainstem were subtracted from the corresponding whole-brain fluorescence signals and analyzed at 1 h, 4 h, 24 h, 7 days, and 14 days after intranasal administration of ICG either as an aqueous solution or encapsulated in ICG-encapsulated PLGA nanoparticles (Figure 4). Quantitative analysis demonstrated that fluorescence intensities in brain regions beyond the OB and brainstem were consistently higher in animals treated with encapsulated ICG than in those receiving aqueous solution of ICG at all measured time points (Figure 4A). This difference likely reflects the more efficient overall brain delivery of ICG-loaded nanoparticles from the nasal cavity, which was also evident in the OB and brainstem. Additionally, the residual whole-brain signal decreased more slowly over time in the group treated with encapsulated ICG, with a statistically significant difference compared to the aqueous ICG group (Figure 4B). Therefore, the more pronounced distribution appears to be mainly attributable to enhanced nose-to-brain transport, while the efficiency of intra-brain distribution likely plays a comparatively minor role. This suggestion is supported by the similar percentages of NIR fluorescence from the compared brain regions, i.e., rat brain areas different from OB and brainstem.

### 2.3. ICG Biodistribution in Peripheral Organs

**Figure 5:**
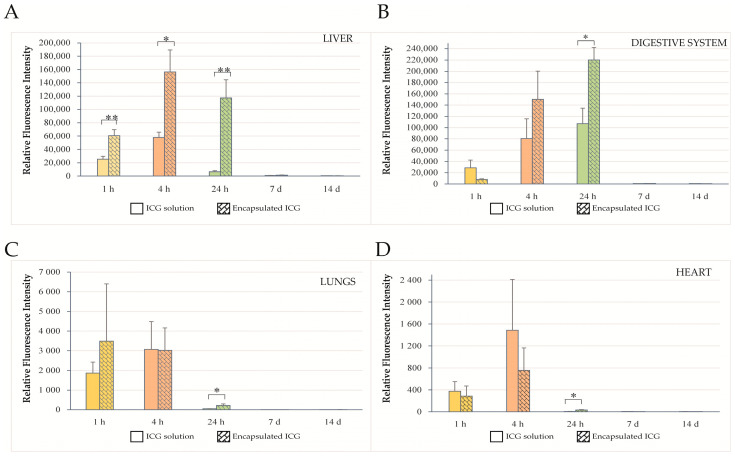
Internal organ biodistribution of intranasally administered ICG. RFI values of ICG fluorescence measured in the liver (**A**), gastrointestinal tract (**B**), lungs (**C**), and heart (**D**) at 1 h, 4 h, 24 h, 7 days, and 14 days after intranasal administration of ICG either as an aqueous solution (solid columns) or as PLGA/fucoidan nanocomposite microparticles (pattern-colored columns). Data are presented as mean ± SEM (*n* = 6). * *p* < 0.05; ** *p* < 0.01.

NIR fluorescence was also detected in several peripheral organs following intranasal administration of ICG either as an aqueous solution or as ICG-encapsulated PLGA nanoparticles (Figure 5). Quantitative analysis demonstrated significantly higher RFI values in the liver of animals treated with ICG-encapsulated PLGA nanoparticles compared with those receiving aqueous ICG during the first 24 h after administration (Figure 5A; *p* < 0.05–0.001; *n* = 6). At 1 h, 4 h, and 24 h, liver fluorescence was markedly elevated in the nanoparticle-treated group, whereas the differences were no longer significant at 7 and 14 days. Similarly, significantly higher RFI values were detected in the gastrointestinal tract at 24 h in animals treated with ICG-encapsulated PLGA nanoparticles (Figure 5B; *p* < 0.05), whereas no significant differences were observed at the remaining time points. In the lungs and heart, significantly increased RFI values were detected at 24 h in the PLGA-ICG group compared with aqueous ICG (Figure 5C,D; *p* < 0.05). At all other time points, fluorescence levels in these organs did not differ significantly between the two groups. Collectively, these findings indicate that encapsulation of ICG within PLGA nanoparticles is associated with delayed elimination.

## 3. Discussion

This study, for the first time, presents evidence that:

(1) ICG rapidly reaches brain structures via both nose-to-brain delivery pathways—the olfactory and the trigeminal routes—enabling fast transport to the olfactory bulbs and brainstem in rats, followed by distribution to other brain regions.
(2) Following intranasal administration at equal doses, ICG encapsulated in PLGA nanoparticles tends to produce stronger and more widespread fluorescence in the whole brain, olfactory bulbs, and brainstem compared with the aqueous formulation.
(3) ICG is a suitable tracer for long-term studies of nose-to-brain delivery, as its NIR emission from the rat brain remains detectable for at least two weeks.
(4) After release from the fucoidan micromatrix, ICG-encapsulated PLGA nanoparticles likely penetrate, at least in part, into the rat brain as intact nanostructures and therefore represent a promising carrier system for nose-to-brain delivery of substances that do not, or only weakly, cross the olfactory mucosa.

The rat brain’s NIR emissions after intranasal administration of ICG in aqueous solution showed a similar temporal profile to that observed in our pilot study, in which a lower dose of ICG was administered as an aqueous solution and monitored over 24 h [31]. However, several notable differences are evident when our data are compared with those reported by Samala et al. (2025) [30] for the same time interval. First, Samala et al. did not register fluorescence in the animal group treated with an ICG suspension at 12 and 24 h after its administration, either in the nostrils or in the brain. Second, the in vivo head imaging presented in that study does not provide details on the specific brain areas that can accumulate ICG, as evidenced by a strong emission signal. Third, at the 3rd hour after intranasal administration of ICG (either as a suspension or incorporated into lipid nanostructures), no detectable brain staining was reported. All these data are illustrated in Figure 6A of [30]. We place particular emphasis on this comparison because, to our knowledge, Samala et al. represents the only other group that has investigated the nose-to-brain delivery of ICG.

Although numerous studies have reported the feasibility of nose-to-brain delivery using PLGA nanoparticles loaded with various therapeutic agents, only a limited number have evaluated the in vivo brain delivery of drug-loaded nanoparticles in detail. Nigam et al. (2019) employed gamma scintigraphy to compare oral, intranasal, and intravenous administration of radiolabeled lamotrigine-loaded PLGA nanoparticles, demonstrating pronounced brain accumulation, reduced peripheral distribution, and a significantly higher C_max_ of lamotrigine in brain tissue following intranasal administration [32]. Similarly, Sharma et al. (2016) [33] reported enhanced brain targeting after intranasal delivery of midazolam-loaded PLGA nanoparticles. Chu et al. (2018) [34] further showed that intranasal administration of temozolomide-loaded PLGA nanoparticles functionalized with anti-EPHA3 resulted in increased brain accumulation and reduced systemic exposure in rats. Comparable improvements in brain targeting and biodistribution following intranasal administration were also described [35,36].

Despite these encouraging findings, the exact mechanisms responsible for the nose-to-brain transport of drug-loaded PLGA nanoparticles remain insufficiently understood. In particular, none of the aforementioned studies systematically investigated the relative contribution of the olfactory and trigeminal pathways, nor did they clarify whether PLGA nanoparticles reach the brain predominantly as intact nanostructures or mainly through drug release at the nasal mucosa followed by secondary transport. This mechanistic gap limits rational optimization of nanoparticle-based intranasal formulations for efficient and targeted brain delivery. In contrast to these reports, the present study provides a more comprehensive in vivo evaluation by simultaneously assessing brain distribution, long-term retention, and the underlying nose-to-brain transport pathways, thereby extending beyond the scope of previously published investigations.

Additionally, in our study, ICG emissions from the liver at 24 h after intranasal administration, whether applied as an ICG solution or as ICG-encapsulated PLGA nanoparticles, were substantially higher than those observed in the lungs. In contrast, in [30], the fluorescence from the lungs at the same time point after administration of ICG-loaded lipid nanostructures was several-fold higher than that from the liver.

The observed differences may be attributed to several factors. First, in our study, fluorescence was measured in excised brains, whereas Samala et al. [30] monitored the entire head signal. Additionally, their registration device was described as an “IVIS (i.e., In Vivo Imaging System) instrument for ex vivo NIR fluorescence” without further technical details [30]. This IVIS system appears to differ from our Pearl Trilogy system, as it employs an excitation wavelength of 710 nm, which excites ICG with an average efficiency of approximately 40%. In contrast, under our experimental conditions, the 785 nm excitation laser achieves nearly 100% excitation efficiency [37]. Furthermore, Samala et al. administered a lower dose of ICG and used a different carrier system (nanostructured lipid carrier). Finally, in some of their presented images, the fluorescence signals from the brain (the target organ) and from the nasal cavity—the administration site, appear to be superimposed, as shown in Figure 6A from [30].

The retention and, as a result, slower absorption of ICG from the gastrointestinal tract, expressed by a stronger fluorescent signal from this part of the rat’s body at 4 and 24 h, and its slow clearance from the liver during the first day after administration of ICG-encapsulated PLGA nanoparticles (Figure 5B), suggest long-term stability of PLGA nanostructures in rats. This assumption is supported by the markedly higher NIR fluorescence observed in the rat liver on the first day after administration of ICG-encapsulated PLGA nanoparticles, compared with the corresponding signal detected after intranasal administration of an ICG solution. The suggested gut–liver transfer of nanoparticles is supported by observations that PLGA nanoparticles can penetrate the intestinal mucosal barrier via microfold (M) cells and subsequently enter the systemic circulation [38]. The higher ICG signal observed in the rat liver following administration with PLGA nanostructures can be attributed to enhanced intestinal absorption of these structures and subsequent hepatic distribution of the nanoparticles. This process may slow hepatic clearance, as the liver is the primary organ responsible for ICG excretion, eliminating the dye via bile without metabolic transformation [39]. Renal elimination of ICG is negligible [39].

ICG does not normally accumulate in the lungs, as it is rapidly eliminated from the blood by the liver [40]. Therefore, its accumulation in the lungs is abnormal. Under our experimental conditions, this can be explained by at least two reasons: i) the capacity of the anion transporter Oatp1b2 in the rat liver is easily saturated compared to the organic anion-transporting polypeptides OATP1B1 and OATP1B3 in humans, and therefore the clearance of high doses of organic anions like ICG dye in rat is less effective [41,42], and ii) short-term exposure to volatile anesthetics such as isoflurane increases the permeability of endothelial cells and may thus enhance ICG penetration into the lungs [43].

Widely used in clinical practice, ICG allows angiographic assessment of the choroidal and retinal circulation, providing high-contrast visualization of the ocular vasculature for both diagnostic and surgical applications [44,45]. In neurosurgical procedures, ICG is routinely administered to visualize deeply located or otherwise inaccessible tumors, enhancing intraoperative delineation of pathological tissue and thereby supporting safer and more complete tumor resection [46]. Although intravenous administration shortly before or on the day of the procedure remains the standard, alternative delivery routes have been studied to a minimal extent. The findings of the present study suggest that intranasal nose-to-brain delivery of ICG may represent a promising approach for neurological imaging, potentially reducing both imaging time and the total dose required. By limiting systemic exposure, this strategy may reduce associated risks, which is particularly advantageous for repeated imaging procedures or in vulnerable patients.

Furthermore, ICG-loaded supramolecular delivery systems, including nanoparticles, liposomes, solid lipid nanoparticles, and dendrimers, enable direct visualization of carrier transport from the nasal cavity to the brain. These imaging approaches facilitate the optimization of formulation and delivery strategies in nose-to-brain drug delivery studies by providing mechanistic insight into carrier migration along neural pathways [47]. In addition, they allow assessment of the effects of particle size, chemical composition, and surface modifications on drug distribution, thereby supporting rational formulation optimization [48].

The employed whole-brain ex vivo imaging technique offers several advantages for evaluating nose-to-brain delivery of ICG. These are: (i) NIR fluorescence from the nasal cavity is not superimposed with the whole brain signal; (ii) it permits visualization of the major transport pathways from the nasal cavity to the brain and measurement of their participation (relative importance) in the studied nose-to-brain delivery process; and (iii) identification with higher spatial resolution of the common brain structures where the dye and neuroactive substances accumulate for further histological studies. Finally, such long-term studies allow monitoring the clearance of labeled structures and molecules, and their subsequent transport from the initial nose-to-brain delivery areas—the OB and brainstem.

## 4. Materials and Methods

All experimental procedures were conducted in accordance with the Guiding Principles for the Care and Use of Laboratory Animals and were approved by the Bulgarian Center for Bioethics and the Ethics Committee for Animal Experiments of Sofia University. The study also complied with the International Guiding Principles for Biomedical Research Involving Animals issued by the Council for International Organizations of Medical Sciences. We have ethical approval from the Bulgarian Food Safety Agency, Ministry of Agriculture, Food and Forestry (No. 381, 12 March 2024) and from the local Ethics Committee of the Faculty of Biology, Sofia University “St. Kliment Ohridski” (Protocol No. 3, 12 February 2024).

Intranasal administration of ICG. Three-month-old Wistar rats were used in the experiments. The animals were divided into two groups: the first weighed 289.55 ± 11.95 g (*n* = 31), and the second 277.30 ± 13.58 g (*n* = 27). The animals were anesthetized using an inhalation anesthetic, consisting of a mixture of isoflurane (Baxter, Sofia, Bulgaria) and oxygen (4% isoflurane for induction, followed by 1.5% isoflurane for maintenance for 5 min), and subsequently injected with Nalgosed (Bioveta, a.s. (C), Ivanovice na Hané, Czech Republic) (2 mg/kg) and Sedin (Vetpharma, Barcelona, Spain) (3.75 mg/kg) before intranasal administration. Both groups received equal doses of ICG. Animals in the first group received a single administration of ICG aqueous solution (64 µg per application) into one nostril. The second group received ICG-encapsulated PLGA nanoparticles (3 mg per application), also into one nostril. Both formulations were applied in the upper 1/3 of the nasal cavity via a venous cannula (G-23). Each group was further subdivided into five subgroups. The brains, nasal cavities, and other organs were isolated and imaged at 1st, 4th, and 24 h, and 7 or 14 days after application, respectively. The anesthetized animals of each group were euthanized gently; the temporal and nasal bones were removed, and the brain was gently excised along the liver, digestive system, heart, and lung. Brains, nasal cavities, and other organs were placed in a Petri dish, and an image was acquired with the Pearl Trilogy Imaging system (LI-COR Biotechnology GmbH, Bad Homburg, Germany) at wavelengths of 800 nm. The brain images were obtained in the ventral plane, whereas the nasal cavity and organs were imaged in the dorsal plane. The first NIR registration was always one hour after ICG administration. RFI values for distribution were normalized to the equal surface of the corresponding area according to the recommendation of the Image Studio v6.0 software, Pearl Trilogy system.

Preparation of ICG-encapsulated PLGA nanoparticles. ICG-encapsulated PLGA nanoparticles were prepared using a double-emulsion solvent-evaporation method in two stages. In the first stage, the inner aqueous phase (W1, 20 mL), containing ICG 0.5% (*w*/*v*), was emulsified into the organic phase (40 mL) comprising PLGA, 0.25% (*w*/*v*), and Span 85, 1% (*w*/*v*), dissolved in dichloromethane (DCM). Homogenization was performed at 21,000 rpm for 3 min (Miccra MiniBatch D-9, MICCRA GmbH, Heitersheim, Germany) to obtain the primary emulsion (W1/O). In the second stage, the primary emulsion was dispersed into 100 mL of the external aqueous phase (W2), which consisted of purified water with 1% (*w*/*v*) polysorbate 20 as the emulsifier. High-speed homogenization at 25,000 rpm for 5 min yielded the W1/O/W2 (Water/Oil/Water) double emulsion system. The nanoemulsion was then mechanically stirred (HS-100D, Witeg Labortechnik GmbH, Wertheim, Germany) at 600 rpm until complete DCM evaporation, allowing solidification of the PLGA droplets.

Preparation of nanocomposite microspheres. Fucoidan served as a transient microscale matrix enabling spray-drying and nasal deposition, dissolving rapidly after administration to release embedded PLGA nanoparticles. Fucoidan microspheres incorporating the formulated PLGA nanoparticles were produced via spray drying using a Büchi Mini Spray Dryer B-290 (Büchi Labortechnik AG, Flawil, Switzerland). Fucoidan was dissolved in the ICG-loaded PLGA nanosuspension, obtained in the previous section, to achieve a concentration of 3% (*w*/*v*). The resulting dispersion was stirred at 300 rpm and spray-dried through a 0.7 mm nozzle under pressurized nitrogen (5 bar) with an aspiration rate of 35 m^3^/h. To obtain microspheres with a mean diameter greater than 5 µm, within the reported optimal size range (5–10 μm) for nasal deposition and potential access to the olfactory region, the process parameters were adjusted as follows: inlet temperature, 160 °C; feed rate, 7.5 mL/min; and gas flow, 600 L/h. The prepared microspheres exhibited a mean diameter of 7.44 ± 1.50 µm with a narrow size distribution, as determined by laser diffraction, placing the majority of particles within the size range reported to be favorable for deposition in the nasal and olfactory region.

Indocyanine green, poly (D, L-lactide-co-glycolide) (PLGA; lactide: glycolide 75:25, Mw 66,000–107,000 g/mol), fucoidan (from Fucus vesiculosus, ≥95%), polysorbate 20 (Mw 1220 g/mol), and Span 85 (Mw 957.49 g/mol) were purchased from Sigma-Aldrich (St. Louis, MO, USA). All other reagents and solvents were of analytical grade and used as provided.

Near-infrared emission. NIR emission was measured using the Pearl Trilogy Small Animal Imaging System (LI-COR Biotechnology GmbH, Bad Homburg, Germany). ICG exhibits Stokes luminescence with excitation in the 750–800 nm range and an emission peak at 830 nm [49]. In this study, ICG fluorescence was activated with a 785 nm laser, and emission was measured at 800 nm. Penetration and distribution in the brain and other structures were assessed by NIR fluorescence intensity relative to the background, presented as the difference between background and fluorescent areas.

Statistical analysis. Statistical analysis was performed using the R statistical programming software, version 4.4.0 (Copyright © 2024, The R Foundation for Statistical Computing). Data are presented as mean ± standard error of the mean (SEM). Differences between groups were assessed using Student’s *t*-test for independent samples. Differences were considered statistically significant at *p* < 0.05. A post hoc power analysis was performed for the primary comparison of nasal retention between aqueous solution and ICG-encapsulated PLGA nanoparticles groups.

## 5. Conclusions

Our results demonstrate that ICG, when loaded into fucoidan microspheres containing formulated PLGA nanoparticles and administered intranasally as a dry powder, can cross the rat nasal mucosal barrier, leading to long-term accumulation in the olfactory bulb and brainstem, with additional distribution to other brain regions. The sustained NIR signal and widespread brain distribution of ICG, combined with the known stability of PLGA nanoparticles and their preferential accumulation in the liver and gastrointestinal tract, strongly suggest that the observed fluorescence reflects penetration of ICG in its nanoparticle form. These findings indicate that PLGA nanoparticles represent a promising platform for brain-directed delivery of non-permeable molecules and supramolecular structures, with potential applications in research, neuroprotection, neuroregeneration, and therapeutic interventions.

## Data Availability

Data available on request.

## Abbreviations

The following abbreviations are used in this manuscript:

CNS	Central nervous system
DCM	Dichloromethane
ICG	Indocyanine green
IVIS	In vivo imaging system
M cells	Microfold cells
NIR	Near-infrared
OB	Olfactory bulbs
OATP	Organic anion-transporting polypeptide
Oatp1b2	Organic anion transporting polypeptide 1b2
PLGA	Poly (D, L-Lactide-co-Glycolide)
RFI	Relative fluorescence intensity
SEM	Standard error of the mean
W1/O/W2	Water/Oil/Water double emulsion system

## References

[1] Frey W.H. (1991). Neurologic Agents for Nasal Administration to the Brain.

[2] Keremidarska-Markova M., Sazdova I., Mladenov M., Pilicheva B., Zagorchev P., Gagov H. (2024). Sirtuin 1 and hormonal regulations in aging. Appl. Sci..

[3] Crowe T.P., Hsu W.H., Talevi A. (2024). Intranasal Delivery of Drugs to the Central Nervous System. CNS Drug Development and Delivery.

[4] Patharapankal E.J., Ajiboye A.L., Mattern C., Trivedi V. (2024). Nose-to-brain (N2B) delivery: An alternative route for the delivery of biologics in the management and treatment of central nervous system disorders. Pharmaceutics.

[5] Hanson L.R., Frey W.H. (2008). Intranasal delivery bypasses the blood–brain barrier, targeting therapeutic agents to the central nervous system and treating neurodegenerative disease. BMC Neurosci..

[6] Alquisiras-Burgos I., González-Herrera I.G., Alcalá-Alcalá S., Aguilera P. (2024). Nose-to-brain delivery of resveratrol, a non-invasive method for the treatment of cerebral ischemia. Drugs Drug Candidates.

[7] Sharma A., Sharma A., Kumar G., Virmani T., Pathak K. (2025). Nano approaches toward CNS drug delivery. Nanomedicine for Neurodegenerative Disorders: Selective Treatment Strategies.

[8] Danielyan L., Schäfer R., von Ameln-Mayerhofer A., Buadze M., Geisler J., Klopfer T., Burkhardt U., Proksch B., Verleysdonk S., Ayturan M. (2009). Intranasal delivery of cells to the brain. Eur. J. Cell Biol..

[9] Lv Z., Li Y., Wang Y., Cong F., Li X., Cui W., Han C., Wei Y., Hong X., Liu Y. (2023). Safety and efficacy outcomes after intranasal administration of neural stem cells in cerebral palsy: A randomized phase 1/2 controlled trial. Stem Cell Res. Ther..

[10] Jiang S., Wang H., Yang C., Feng F., Xu D., Zhang M., Xie M., Cui R., Zhu Z., Jia C. (2024). Phase 1 study of safety and preliminary efficacy of intranasal transplantation of human neural stem cells (ANGE-S003) in Parkinson’s disease. J. Neurol. Neurosurg. Psychiatry.

[11] Crowe T.P., Hsu W.H. (2022). Evaluation of recent intranasal drug delivery systems to the central nervous system. Pharmaceutics.

[12] Nguyen T.T., Duong V.A. (2025). Advancements in nanocarrier systems for nose-to-brain drug delivery. Pharmaceuticals.

[13] Guo Y., Tang Y., Su Y., Sun D. (2024). Influencing factors of particle deposition in the human nasal cavity. Laryngoscope Investig. Otolaryngol..

[14] Vishnumurthy P., Radulesco T., Bouchet G., Regard A., Michel J. (2025). Computational fluid dynamics approach for direct nose-to-brain drug delivery: A systematic review and meta-analysis. J. Pers. Med..

[15] Gänger S., Schindowski K. (2018). Tailoring formulations for intranasal nose-to-brain delivery: A review on architecture, physicochemical characteristics and mucociliary clearance of the nasal olfactory mucosa. Pharmaceutics.

[16] Pandey S., Nainwal N., Negi T., Lohar A., Kumar S., Kumar S., Bisht A. (2025). Nose-to-brain delivery of microcarrier in the treatment of neurodegenerative diseases. J. Appl. Pharm. Sci..

[17] Montegiove N., Calzoni E., Emiliani C., Cesaretti A. (2022). Biopolymer nanoparticles for nose-to-brain drug delivery: A promising approach for the treatment of neurological diseases. J. Funct. Biomater..

[18] Alghareeb S., Asare-Addo K., Conway B., Adebisi A. (2024). PLGA nanoparticles for nasal drug delivery. J. Drug Deliv. Sci. Technol..

[19] Huang Q., Chen X., Yu S., Gong G., Shu H. (2024). Research progress in brain-targeted nasal drug delivery. Front. Aging Neurosci..

[20] Aghaei Delche N., Kheiri R., Ghorbani Nejad B., Sheikhi M., Razavi M.S., Rahimzadegan M., Salmasi Z. (2023). Recent progress in intranasal PLGA-based drug delivery for neurodegenerative disease treatment. Iran. J. Basic Med. Sci..

[21] Su Y., Zhang B., Sun R., Liu W., Zhu Q., Zhang X., Wang R., Chen C. (2021). PLGA-based biodegradable microspheres in drug delivery: Recent advances in research and application. Drug Deliv..

[22] Sharma S., Tyagi A., Dang S. (2023). Nose-to-brain delivery of transferrin-conjugated PLGA nanoparticles for clonidine. Int. J. Biol. Macromol..

[23] Salem H., Ali A., Rabea Y., Abo El-Ela F., Khallaf R. (2023). Optimization and appraisal of chitosan-grafted PLGA nanoparticles for boosting pharmacokinetic and pharmacodynamic effects of duloxetine HCl using Box–Behnken design. J. Pharm. Sci..

[24] Chan J.M., Zhang L., Yuet K.P., Liao G., Rhee J.W., Langer R., Farokhzad O.C. (2009). PLGA–lecithin–PEG core–shell nanoparticles for controlled drug delivery. Biomaterials.

[25] Wang Z.H., Wang Z.Y., Sun C.S., Wang C.Y., Jiang T.Y., Wang S.L. (2010). Trimethylated chitosan-conjugated PLGA nanoparticles for drug delivery to the brain. Biomaterials.

[26] Saren B.N., Mahajan S., Aalhate M., Kumar R., Chatterjee E., Maji I., Gupta U., Guru S.K., Singh P.K. (2024). Fucoidan-mediated targeted delivery of dasatinib-loaded nanoparticles enhances apoptosis in triple-negative breast cancer. Colloids Surf. B Biointerfaces.

[27] Lim Z.Y., Mohan S., Balasubramaniam S., Ahmed S., Siew C., Shelat V.G. (2023). Indocyanine green dye and its application in gastrointestinal surgery. World J. Gastrointest. Surg..

[28] Marasini R., Aryal S. (2022). Indocyanine-type infrared-820 encapsulated polymeric nanoparticles for photothermal cancer therapy. ACS Omega.

[29] Alius C., Oprescu S., Balalau C., Nica A.E. (2018). Indocyanine green-enhanced surgery: Principles, clinical applications and future research directions. J. Clin. Investig. Surg..

[30] Samala S., Mhaske A., Shukla R. (2025). Intranasal apocynin-loaded nanostructured lipid carriers for Alzheimer’s disease therapy. J. Drug Deliv. Sci. Technol..

[31] Mishonova M., Koceva L., Pilicheva B., Zagorchev P., Eftimov P., Gagov H., Sazdova I. (2025). Nose-to-Brain Delivery of Indocyanine Green in Rats. Proc. Bulg. Acad. Sci..

[32] Nigam K., Kaur A., Tyagi A., Nematullah M., Khan F., Gabrani R., Dang S. (2019). Nose-to-brain delivery of lamotrigine-loaded PLGA nanoparticles. Drug Deliv. Transl. Res..

[33] Sharma D., Sharma R.K., Bhatnagar A., Nishad D.K., Singh T., Gabrani R., Sharma S.K., Ali J., Dang S. (2016). Nose-to-brain delivery of midazolam-loaded PLGA nanoparticles: In vitro and in vivo investigations. Curr. Drug Deliv..

[34] Chu L., Wang A., Ni L., Yan X., Song Y., Zhao M., Sun K., Mu H., Liu S., Wu Z. (2018). Nose-to-brain delivery of temozolomide-loaded PLGA nanoparticles functionalized with anti-EPHA3 for glioblastoma targeting. Drug Deliv..

[35] Shah P., Sarolia J., Vyas B., Wagh P., Ankur K., Kumar M.A. (2021). PLGA nanoparticles for nose-to-brain delivery of clonazepam: Formulation, optimization by 32 factorial design, in vitro and in vivo evaluation. Curr. Drug Deliv..

[36] Cayero-Otero M.D., Gomes M.J., Martins C., Álvarez-Fuentes J., Fernández-Arévalo M., Sarmento B., Martín-Banderas L. (2019). In vivo biodistribution of venlafaxine–PLGA nanoparticles for brain delivery: Plain vs. functionalized nanoparticles. Expert Opin. Drug Deliv..

[37] Almarhaby A.M., Lees J.E., Bugby S.L., McKnight W.R., Alqahtani M.S., Jambi L.K., Perkins A.C. (2019). Characterisation of a near-infrared (NIR) fluorescence imaging systems intended for hybrid gamma-NIR fluorescence image guided surgery. J. Instrum..

[38] Yen Y.W., Lee Y.L., Yu L.Y., Li C.E., Shueng P.W., Chiu H.C., Lo C.L. (2023). Fucoidan/chitosan layered PLGA nanoparticles with melatonin loading for inducing intestinal absorption and addressing triple-negative breast cancer progression. Int. J. Biol. Macromol..

[39] Delgado-Tirado S., Gonzalez-Buendia L., Kim L.A., Albert D.M., Miller J.W., Azar D.T., Young L.H. (2021). Indocyanine Green Angiography. Albert and Jakobiec’s Principles and Practice of Ophthalmology.

[40] Caimano M., Bianco G., Coppola A., Marrone G., Agnes S., Lai Q., Spoletini G. (2024). Indocyanine green clearance tests to assess liver transplantation outcomes: A systematic review. Int. J. Surg..

[41] van de Steeg E., van Esch A., Wagenaar E., Kenworthy K.E., Schinkel A.H. (2013). Influence of human OATP1B1, OATP1B3, and OATP1A2 on the pharmacokinetics of methotrexate and paclitaxel in humanized transgenic mice. Clin. Cancer Res..

[42] Frisch K., Keiding S. (2020). Use of Indocyanine Green (ICG) in Hepatology. Adv. Res. Gastroentero Hepatol..

[43] Noorani B., Chowdhury E.A., Alqahtani F., Ahn Y., Nozohouri E., Zoubi S., Patel D., Wood L., Huang J., Siddique M.B. (2023). Effects of Volatile Anesthetics versus Ketamine on Blood–Brain Barrier Permeability via Lipid-Mediated Alterations of Endothelial Cell Membranes. J. Pharmacol. Exp. Ther..

[44] Wu Y., Yang X., Zhang C. (2025). CACA guidelines for fluorescence-guided surgery with indocyanine green (oral cancer section). Holist. Integr. Oncol..

[45] Gelişken F. (2024). Indocyanine Green Angiography. Turk. J. Ophthalmol..

[46] Mansour H.M., Shah S., Aguilar T.M., Abdul-Muqsith M., Gonzales-Portillo G.S., Mehta A.I. (2024). Enhancing Glioblastoma Resection with NIR Fluorescence Imaging: A Systematic Review. Cancers.

[47] Veronesi M.C., Alhamami M., Miedema S.B., Yun Y., Ruiz-Cardozo M., Vannier M.W. (2020). Imaging of intranasal drug delivery to the brain. Am. J. Nucl. Med. Mol. Imaging.

[48] Almahmoud A., Parekh H.S., Paterson B.M., Tupally K.R., Vegh V. (2024). Intranasal delivery of imaging agents to the brain. Theranostics.

[49] Miwa M. (2010). The principle of ICG fluorescence method. Open Surg. Oncol. J..

